# 

**Published:** 1888-06-02

**Authors:** 


CONTENTS.

**Speakers and Speech** - - - - - - - 133

**Points for Preachers, Churchworkers, and Pressmen.**—A Hint to Churchwardens—The Hospitals' Week—To the Clergy and Ministers - - - - - 134

**The Vinnicombe Bequest: A Narrative of Hospital Work and Hospital Wants**—Mr. Timberlake's Promise—Mr. Vinnicombe gives Trouble—Dr. Rowe—Vinnicombe's Bequest—A Few Letters!—Death or Debt—What have the Hospitals done for Me?—What does the Nation do?—Do Hospitals Pauperise the Patients?—The Voluntary System—Hospital Sunday—A Word to Life Insurance Companies—A Blot on the System—Which should get it?—What are Nervous Diseases?—Ask if you would Receive—Knowledge brings Respect—A Case for the Hospital—Miss Kate Verity—Within the Hospital Doors—Looking for Russell—The Hospital for Nervous Diseases—Why Hospitals should be Attractive—What Need is there for Special Hospitals?—In the Out-patients' Room—Quick Work!—The Case of John Harvey—John Harvey in the Ward—The Perfection of Philanthropy—A Terrible Case—The Man with the Abscess on his Brain—Death in Life—Aphasia—Massage and Electricity—The Cure for Neuralgia—Discipline and Work A Case of Convulsions—The Patient with the Helmet—The Cost of an Operation—Russell at Last—The Electrical Room, etc., etc. - 135

**Christianity and the Sick** - - - - - - 151

**Facts and Figures** - - - - - - 152

**A Year's Work in the Hospitals and Medical Charities of London** - - - - - 153

**The Nursing Mirror**

